# Imported Eosinophilia in Migrants from Endemic Areas in Spain

**DOI:** 10.3390/tropicalmed11010020

**Published:** 2026-01-11

**Authors:** Laura Niño-Puerto, Belén Vicente, Juan Hernández-Goenaga, Javier Pardo Lledías, Juan Luis Muñoz Bellido, Moncef Belhassen-García, Antonio Muro

**Affiliations:** 1 Infectious and Tropical Diseases Research Group (e-INTRO), Biomedical Research Institute of Salamanca Research Centre for Tropical Diseases at the University of Salamanca (IBSAL-CIETUS), 37007 Salamanca, Spainama@usal.es (A.M.); 2 Infectious Diseases Unit, Internal Medicine and Microbiology, University Hospital of Salamanca, Biomedical Research Institute of Salamanca, Research Centre for Tropical Diseases at the University of Salamanca (IBSAL-CIETUS), 37007 Salamanca, Spain; 3 IDIVAL (Instituto de Investigación Valdecilla), Internal Medicine Service, Hospital Marqués de Valdecilla, University of Cantabria, 39005 Santander, Spain

**Keywords:** eosinophilia, parasitic infections, helminthiasis, immigrants

## Abstract

Eosinophilia is a valuable biomarker for estimating the likelihood of parasitic infection in immigrants from tropical or subtropical regions. This study aimed to evaluate the frequency and etiology of imported eosinophilia in patients attending the Tropical Medicine Unit (TMU) of Salamanca, Spain, between 2008 and 2023. A total of 773 immigrant patients were assessed: 450 (58.2%) from Africa, 306 (39.6%) from Latin America, and 17 (2.2%) from Asia. Eosinophilia was observed in 338 patients (43.7%), of whom 15 (4.4%) had noninfectious causes. Among the remaining 323 evaluated for infections, 171 (52.9%) presented with relative eosinophilia and 152 (47.1%) presented with absolute eosinophilia. A specific diagnosis was reached in 49.2% of the cases, most commonly filariasis (12.1%), strongyloidiasis (9.9%), and schistosomiasis (4.6%): 58 patients had coinfections. In conclusion, eosinophilia is common among migrants and represents a valuable biomarker for helminthiasis. Despite protocolized evaluation, nearly half of the cases remain undiagnosed. The most frequent etiologies were filariasis, strongyloidiasis, and schistosomiasis, with African patients having the highest probability of diagnosis. Improved diagnostic approaches, including tests for less common parasites, may reduce uncertainty and enhance clinical management.

## 1. Introduction

Eosinophilia is a common hematological alteration characterized by an increase in the number of circulating eosinophils [1], which can be triggered by various conditions, including drug use and immune-allergic, endocrinological, and neoplastic diseases [2]. Additionally, this alteration can also be caused by infections, most often helminthic infections, but occasionally by some bacterial, viral, fungal, or protozoal infections [3,4,5]. Although reference values vary among different authors [3,6], the consensus document of the Spanish Society of Tropical Medicine and International Health (in Spanish, abbreviated as SEMTSI) defines imported eosinophilia as an increase in the number of eosinophils in peripheral blood above 0.45 × 10^9^ cells/L [6].

Imported eosinophilia is frequently attributable to parasitic infections, especially those caused by helminth infections, among immigrants or travelers from tropical or subtropical regions. In different series, a parasitic etiology has been identified in more than half of the reported cases [7,8]. Several studies conducted in Spain have shown that parasitic infections are a common cause of eosinophilia, and that a considerable proportion of patients present multiple parasitic infections, most often due to *Strongyloides stercoralis, Schistosoma* spp. and *hookworms* [9,10,11]. In addition to these national data, recent international data from refugee and migrant cohorts in Europe and North America have confirmed that eosinophilia remains a frequent finding in these populations and is widely used as a screening marker for underlying helminth infections, with current guidelines recommending systematic evaluation and follow-up in newly arrived migrants [12,13,14].

A minority of eosinophilia cases, known as autologous eosinophilia, are attributed to allergic reactions, immunological conditions, neoplasms, and other noninfectious factors [3,6,11]. However, a substantial proportion of cases do not have an identified etiology, as reported in several series, highlighting that the underlying cause of eosinophilia often remains unknown [1,15]. These findings underscore the need for a deeper understanding of the underlying factors associated with eosinophilia in tropical and subtropical regions, which contributes to improved diagnostic strategies and more effective clinical management of this condition.

For such cases, a clear consensus regarding the diagnostic protocol to be applied has not yet been established. The literature consistently refers to cases without an etiological cause for imported eosinophilia [16], despite multiple analyses typically including complete blood count, biochemical study, chest X-ray, urine sediment, stool examination, and other tests for the detection of the most common parasites (*Schistosoma* spp., *Taenia* spp., filarias, and geohelminths) [6]. Previous studies have considered relative and absolute eosinophilia as good indicators to facilitate the diagnosis of helminthiasis in immigrants and travelers from endemic areas. In these situations, it is necessary to rule out this potential parasitosis as the cause of eosinophilia since it is related to long-term morbidity and mortality.

On the basis of the aforementioned factors, this study aims to describe the frequency and etiologies of imported eosinophilia in patients attending the Tropical Medicine Unit of the Hospital of Salamanca (Spain).

## 2. Materials and Methods

### 2.1. Study Population and Data Collection

This study was conducted at the Tropical Medicine Unit (TMU)—Complejo Asistencial Universitario de Salamanca (CAUSA), Salamanca (Spain). The prevalence and causes of eosinophilia in immigrant patients from tropical and subtropical areas were retrospectively evaluated. The inclusion criteria were patients who (1) were evaluated at the TMU clinic from 2008–2023, (2) originated from tropical or subtropical areas, and (3) whose complete blood test results were available.

Patients were evaluated for different reasons, including routine screening, clinical suspicion of infection, or other medical conditions. In addition, routine studies, including tests for HIV, syphilis, HBV, HCV, and HTLV, were carried out according to the clinical suspicion of the physician. Other medically relevant data, such as age, sex, medical history, allergies, and medication use, were extracted from patients’ medical records to determine their clinical characteristics. Figure S1 shows the diagnostic protocol applied to immigrant patients at the TMU.

### 2.2. Definitions

The values used as a reference for comparison among the patients included in this study are those defined by SEMTSI [9,17]. Specifically, eosinophilia is defined as an increase in peripheral blood eosinophilic leukocytes to more than ≥0.45 × 10^9^ eosinophils/L, with mild absolute eosinophilia defined as 0.45–0.99 × 10^9^ eosinophils/L, moderate eosinophilia 0.99–2.99 × 10^9^ eosinophils/L, and severe eosinophilia ≥ 3.00 × 10^9^ eosinophils/L. Cases with direct parasitological evidence were classified as confirmed infections, whereas those with only a positive serology above the manufacturer’s or validated in-house cutoff were classified as possible infections based on a positive serological result. An isolated positive serology for *Strongyloides* spp. was considered diagnostic due to the parasite’s autoinfective cycle. *Screening* was defined as the routine evaluation of asymptomatic immigrants, usually performed as part of systematic health assessments during general medical check-ups. *Clinical suspicion* referred to the evaluation of immigrants presenting clinical symptoms or laboratory abnormalities suggestive of parasitic infection. *Others* included cases in which immigrants were evaluated for follow-up purposes or when information regarding the indication for testing was incomplete.

### 2.3. Laboratory Data

Indirect parasitological evaluations included commercial serological tests for *Taenia solium* (NovaTec Immundiagnostica GmbH. NovaLisa^®^ *Taenia solium* IgG, Dietzenbach, Germany), and previously validated homemade serological enzyme-linked immunosorbent assays (ELISAs) developed by our research group, which use antigens from adult whole worms of the species *Dirofilaria immitis* [18] and *Schistosoma bovis* [19,20], excretion/secretion antigens of *Fasciola hepatica* [21], and somatic larvae antigens of *Strongyloides venezuelensis* for the diagnosis of filariasis, schistosomiasis, fasciolosis, and strongyloidiasis, respectively. Direct parasitological tests involved the examination of three concentrated stool samples per patient collection on consecutive days and terminal urine microscopy. In particular cases (i.e., patients from sub-Saharan Africa), a Knott test for microfilaremia and/or skin snips were also used.

### 2.4. Statistical Analysis

A descriptive analysis was conducted for both quantitative and qualitative variables. Quantitative variables with a normal distribution are expressed as the means and standard deviations (SDs), whereas non-normally distributed variables, such as the eosinophil count, are expressed as medians and interquartile ranges (IQRs). A χ^2^ test or Fisher’s exact test (when appropriate) was used to evaluate the associations between categorical variables (e.g., region of origin and final diagnosis and region of origin and degree of eosinophilia). Statistical significance was considered at a *p* value < 0.05. The data were analyzed via the SPSS software package, version 26 (SPSS, Chicago, IL, USA).

### 2.5. Ethics Approval

For this study, medical data from patients who attended the TMU were used. In accordance with privacy laws, these data were anonymized by assigning a numerical code. All procedures described here were carried out in accordance with the Declaration of Helsinki, revised in 2024 [22]. The study protocol was approved by the Ethics Committee of CAUSA (CEIm PI 2023/10 1448).

## 3. Results

### 3.1. Study Population

**Figure 1:**
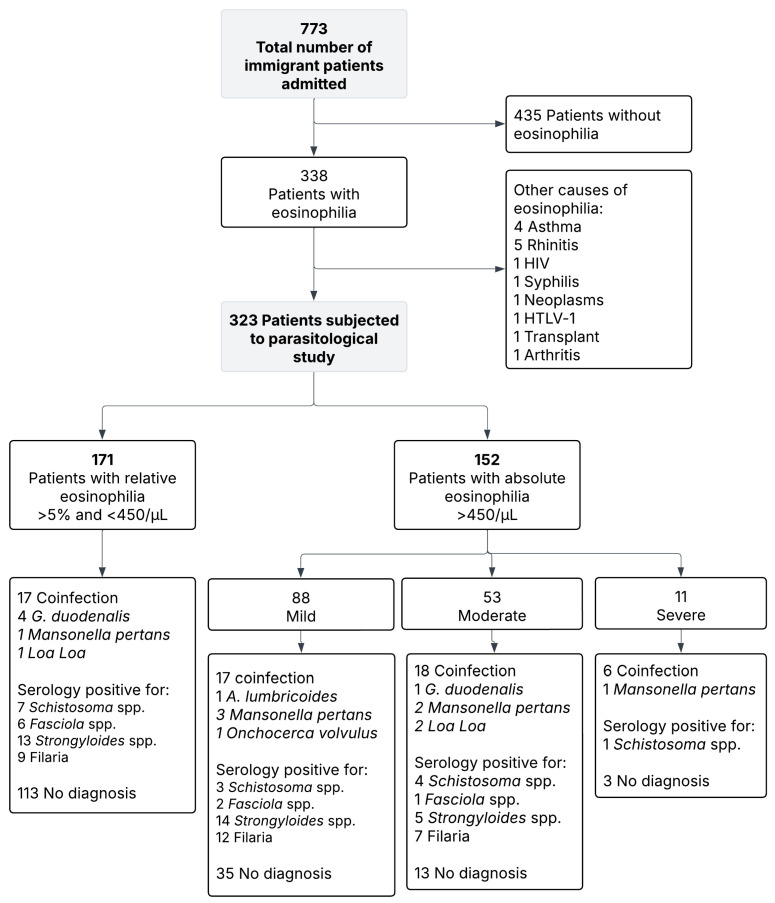
Flowchart of immigrant patients included in the study.

This study included 773 patients from tropical and subtropical areas. The flow chart of the patient profile is shown in Figure 1. Most patients were from sub-Saharan Africa (371/773, 48%) or Latin America (306/773, 39.6%), with the remainder from North Africa (79/773, 10.2%) or Asia (17/773, 2.2%). The most common countries of origin were Equatorial Guinea (240, 31%), Bolivia (100, 12.9%), and Morocco (73, 9.4%). The mean age was 33.6 ± 13.2 years, and the male-to-female ratio was 0.5:1. The countries of origin of all patients are illustrated in Figure S2.

### 3.2. Frequency of Eosinophilia

Eosinophilia was detected in 338 of the 773 patients (43.7%). Fifteen cases (4.4%) were attributable to noninfectious causes, including rhinitis (1.5%), asthma (1.2%), HIV, syphilis, neoplasm, HTLV-1, transplant, and arthritis (0.3% each). The remaining 323 patients were classified as having relative eosinophilia (171, 52.9%) or absolute eosinophilia (152, 47.1%). Among the latter, 88 (57.9%) had mild, 53 (34.9%) had moderate, and 11 (7.2%) had severe eosinophilia. Among the 323 patients with eosinophilia, the median absolute eosinophil count was 0.41 × 10^9^/L (IQR 0.236–0.820). Patients with relative eosinophilia (n = 171) had a median of 0.241 × 10^9^/L (IQR 0.140–0.350), while those with absolute eosinophilia (n = 152) had 0.830 × 10^9^/L (IQR 0.585–1.475).

Among patients with eosinophilia (n = 323), the main reasons for evaluation were general screening in 217 patients (67.2%), clinical suspicion in 101 patients (31.3%), and other causes in 5 patients (1.5%) (Table 1). When stratified by region, screening was most common among immigrants from North Africa (16/17, 94.1%) and Sub-Saharan Africa (148/179, 82.7%), whereas clinical suspicion predominated among Latin American immigrants (71/123, 57.7%) (Table 1). Statistical analysis confirmed that the distribution of reasons for evaluation differed significantly by region (χ^2^ = 90.5, *p* < 0.001). When comparing regions, the prevalence of absolute eosinophilia was significantly greater among Sub-Saharan African patients than among those from Latin America (54.2% vs. 35%, *p* = 0.04). No significant differences were observed in relative eosinophilia among the regions.

### 3.3. Etiologies of Eosinophilia

**Figure 2:**
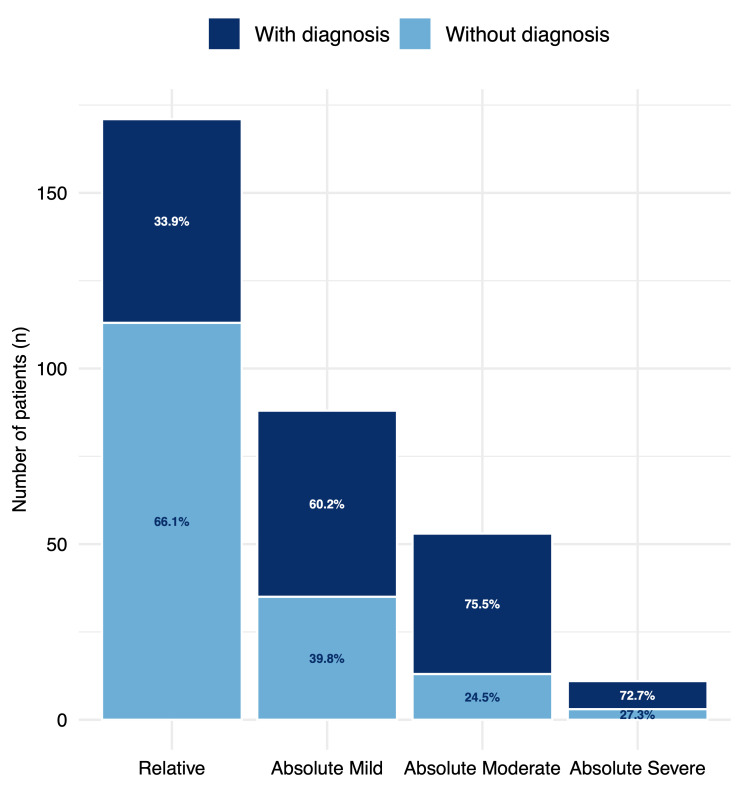
Number of patients with and without a confirmed or possible parasitic diagnosis according to degree of eosinophilia (relative, mild, moderate, and severe).

A specific diagnosis was established in 159 of 323 patients (49.2%), whereas no cause was identified in 164 (50.8%). Eosinophil counts were significantly greater in patients with a diagnosis than in undiagnosed patients (0.951 × 10^9^/L vs. 0.464 × 10^9^/L, *p* < 0.05). Parasitic infections (confirmed or possible) accounted for the majority of diagnoses, with higher confirmation rates in absolute vs. relative eosinophilia (101/152, 63.5% vs. 58/171, 36.5%; *p* < 0.05). A greater proportion of diagnoses was observed in patients referred for screening in consultation (122/217, 56.2%) than in those not screened at the consultation (35/100, 35.0%) (χ^2^ = 12.95, *p* = 0.002). In absolute eosinophilia, filariasis was the most frequent parasitic aetiology (27.7%), followed by strongyloidiasis (18.8%) and schistosomiasis (7.9%). In terms of relative eosinophilia, strongyloidiasis predominated (22.4%), followed by filariasis (19%). Table S3 details the distribution of parasitic infections according to the degree of eosinophilia. Of the 39 patients classified as having filarial infections, 11 had species-specific diagnoses confirmed by direct methods *(Mansonella perstans*, *Loa loa* or *Onchocerca volvulus*), whereas 28 were categorized as ‘filarias’ based solely on positive serology (Table 2). Other analyses were performed, but no significant association with eosinophilia was found. Figure 2 shows the proportion of diagnosed patients according to the type of eosinophilia.

### 3.4. Symptoms in Immigrant Patients

Asymptomatic presentation was observed in nearly half of the patients, with the highest proportions among immigrants from North Africa (65%) and Latin America (55%) (Table 1), and was common across all parasitic infections, including filariasis (55%), schistosomiasis (46%), strongyloidiasis (42%), and fascioliasis (38%) (Table S1). Asymptomatic cases were similarly common among patients with a confirmed or possible parasitic diagnosis and those without (46% vs. 51%; χ^2^ = 6.58, *p* = 0.037; Table S2).

Pruritus was the most frequent symptomatic feature among diagnosed patients (17% vs. 8% in undiagnosed individuals). Eosinophilia itself was recorded as the main reason for consultation in 8% of patients, mostly among Latin American immigrants. The distributions of pruritus and eosinophilia also differed significantly by type of parasitic infection (χ^2^ = 71.9, *p* = 0.014).

Gastrointestinal manifestations, including abdominal pain and diarrhea, were reported in 19% of the patients overall, with abdominal pain being particularly frequent in patients with fascioliasis (25%) and diarrhea being most common in patients with intestinal parasite infections (43%). Cutaneous symptoms occurred in 14% of patients, mainly in Sub-Saharan African immigrants, whereas less frequent symptoms—including fever (4%), ocular complaints (2%), respiratory (1%), genitourinary (0.3%), and unspecific manifestations (2%)—were distributed across regions. The distribution of all symptoms differed significantly across regions (χ^2^ = 56.7, *p* < 0.001), indicating that symptom profiles vary by geographic origin.

### 3.5. Coinfections

Among the 159 patients with a confirmed or possible parasitic diagnosis, 58 (36.5%) presented with coinfections. The most frequent combinations were *Strongyloides* spp. plus filaria (27, 46.6%), *Schistosoma* spp. plus *Strongyloides* spp. plus filaria (11, 19.0%), and *Schistosoma* spp. plus *Strongyloides* spp. (6, 10.3%). Most of these associations were identified on the basis of serological results, which may include some degree of cross-reactivity among helminth antigens.

**Table 2:** Confirmed or possible parasitic diseases in immigrants by area of origin. Tropical Medicine Unit, Complejo Asistencial Universitario de Salamanca, Salamanca, Spain, 2008–2023.

	Sub-Saharan Africa, n %		North Africa, n %		Latin America, n %		Asia, n %		All Subjects, n %	
	n = 179		n = 17		n = 123		n = 4		N = 323	
Positive serological test for strongyloidiasis	17	9.5	1	5.9	13	10.6	1	25	32	9.9
Positive serological test for schistosomiasis	11	6.1	0		3	2.4	1	25	15	4.6
Positive serological test for fasciolasis	4	2.2	0		5	4.1	0		9	2.8
Intestinal parasite	3	1.7	1	5.9	2	1.6	0		6	1.9
Positive serological test for filariasis	16	8.9	4	23.5	8	6.5	0		28	8.7
*Mansonella pertans*	7	3.9	0		0		0		7	2.2
*Loa Loa*	3	1.7	0		0		0		3	0.9
*Onchocerca volvulus*	1	0.6	0		0		0		1	0.3
Coinfection	45	25.1	2	11.8	11	8.9	0		58	18.0
No parasitic disease diagnosed	72	40.2	9	52.9	81	65.9	2	50	164	50.8

### 3.6. Geographic Distribution of Parasitic Infections

Among sub-Saharan African patients, the leading diagnosis was filariasis (15.1%), followed by strongyloidiasis (9.5%) and schistosomiasis (6.1%), with approximately one-third of filarial cases confirmed by direct parasitological methods and the remainder classified as possible infections based on serology alone. Among Latin American patients, *Strongyloides* spp. (10.6%) were the most common, followed by filariasis (6.5%) and *Fasciola* spp. (4.1%). Among Asian patients, one case each of strongyloidiasis and schistosomiasis was identified. Overall, parasitic infections were more common in sub-Saharan African patients than in the other groups (*p* = 0.03). Within the study cohort eosinophilia was identified in 59.8% of sub-Saharan African patients, 47.1% of North African patients, 34.1% of Latin American patients, and 50% of Asian patients. Table 1 and Figure S2 summarize the demographic characteristics of the study population.

## 4. Discussion

Currently, there are over 280 million people living as international immigrants worldwide [23]. In Spain, immigrants constitute 14.6% of the population, a proportion that has steadily increased in recent decades and has been accompanied by an increase in imported infections. Although most immigrants are healthy adults, some individuals, particularly those from resource-limited regions, may carry asymptomatic latent infections that could result in chronic illness and long-term complications [14,24]. In line with this, diseases associated with eosinophilia are frequently asymptomatic or present with nonspecific symptoms, as reported in other migrant cohorts and guidelines [9,14]. The symptomatic presentation in patients in our study was variable, with nearly half showing no clinical manifestations at the time of evaluation. When symptoms were present, pruritus and gastrointestinal complaints such as abdominal pain and diarrhea were the most frequently observed symptoms. Pruritus is particularly associated with helminth infections such as strongyloidiasis, schistosomiasis, and filariasis and may present as generalized itch or as specific cutaneous manifestations such as *larva currens* or urticarial lesions [14,25]. Gastrointestinal symptoms are common in infections with intestinal helminths and protozoa and may include abdominal pain, diarrhea, and, less commonly, nausea or vomiting [14].

Eosinophilia is a frequent finding among travelers and immigrants from tropical and subtropical regions, with a prevalence ranging from 3% to 50%, depending on the region of origin and population characteristics (e.g., mean age, sex, and socioeconomic status) [17]. Elevated eosinophil levels are most commonly associated with parasitic infections, particularly filariasis, strongyloidiasis, and schistosomiasis, but may also occur in less frequent helminth infections, such as toxocariasis [26]. Although *Giardia duodenalis* is not classically associated with eosinophilia, recent evidence [27] has reported eosinophilia in a proportion of patients with gastrointestinal symptoms.

In our study, the proportion of patients with eosinophilia (43.7%) was greater than that reported in other studies [9,10,14,28]. This could be due to several factors: (i) we analyzed diverse samples of patients from different tropical and subtropical regions, which included a greater proportion of African patients than did previous studies; (ii) all patients analyzed in the present study were immigrants, while most mentioned studies also included travelers; (iii) hematologic reference intervals may vary between populations, as reported in African cohorts [29]; (iv) the risk of selection bias inherent to retrospective designs and heterogeneous referral patterns (screening vs. targeted evaluation) may have overestimated the proportion of eosinophilia observed in our cohort, as previously described in similar migrant cohorts [5,30]. Therefore, these findings should not be interpreted as providing true prevalence estimates in the general migrant population, but rather as describing the caseload of a referral tropical medicine unit.

With respect to the different types of eosinophilia reported here, relative eosinophilia was present in 53% of the patients, and absolute eosinophilia was found in 47% of the patients, most of whom were patients originating from sub-Saharan Africa, who were also more frequently diagnosed with helminthiasis. This finding aligns with previous studies and clinical expert consensus documents on imported diseases, highlighting the relevance of absolute eosinophilia as a biomarker for helminthiasis [6,14,31]. Absolute eosinophilia is widely recognized in clinical practice as a reliable biomarker of helminthiasis, which is consistent with our findings. Indeed, in this study, an etiological diagnosis was established more frequently for those patients with absolute eosinophilia. However, we cannot exclude the possibility that patients with relative eosinophilia may have helminthiasis, given that relative eosinophilia has also been shown to be a biomarker of helminthiasis in both children and adults [28,32,33].

The differential diagnosis of eosinophilia is broad, and screening is frequently performed in hospitals and clinics where immigrant patients are admitted [34]. By applying the systematic diagnostic protocol established in our hospital in 2008, we identified the cause of eosinophilia in 52% of all patients, which, compared with other studies [10,17,33], represents a lower percentage. This result may be because, in the previously mentioned studies, African patients accounted for more than 75% of the cases, whereas they represented 60.7% of our sample. In contrast, studies based on a wider variety of participants, including travelers and immigrants from different regions, primarily Latin America and Africa [4,9,35], have reported results similar to ours, with parasitic infections being detected in approximately 65% of cases. This highlights the importance of applying a differential diagnostic protocol on the basis of the patient’s origin, as demonstrated in several studies [6,22,25,38^36^].

In general, the probability of eosinophilia being caused by parasitic helminth infections is greater in patients originating from resource-limited countries [37], with filariasis, schistosomiasis, and geohelminthiasis being the most common infections in patients from Africa. Conversely, hookworm and strongyloidiasis account for almost 100% of cases in immigrant patients from Latin America [3,7]. Our results are consistent with these findings, as filariasis was the most frequent diagnosis in African patients. However, a substantial proportion of filarial infections in our cohort were classified as possible on the basis of serology alone, which may overestimate the contribution of filariasis, particularly in migrants from regions where current transmission is low. Moreover, strongyloidiasis was found to be the most prevalent helminth infection among patients from Latin America.

Apart from the infections caused by a single parasite, the prevalence of coinfections in our cohort was high, with *Strongyloides stercoralis* plus filariasis being the most frequently detected combination, followed by associations including *Schistosoma* spp. Nevertheless, reliance on serological techniques in a retrospective design does not allow for an accurate determination of the actual infection. Such limitations are well recognized in the serologic diagnosis of helminth infections, as antibody-based assays show variable specificity, frequent cross-reactivity among helminths, an inability to differentiate between past exposure and current infection, and test performance varies according to the timing of sample collection [5,14,36,38]. These factors complicate interpretation, particularly in populations from endemic areas, where seropositivity may reflect prior exposure rather than ongoing infection. For this reason, most serology-based diagnoses in our study should be considered as possible rather than confirmed infections, especially in the case of filarial and coinfection patterns. Current guidelines therefore recommend interpreting serological findings in conjunction with clinical, epidemiological, and direct diagnostic data rather than as standalone evidence of active infection [14,39].

Importantly, despite following a structured clinical diagnostic protocol, more than half of the patients in our cohort remained without an identified etiology. This suggests that other factors not considered in this study may induce eosinophilia and parasitic infections, highlighting the complexity and diversity of potential triggers for eosinophilia in immigrant patients. The absence of a definite diagnosis in a significant proportion of our cohort emphasizes the need for a thorough and continuous evaluation of the underlying causative factors to fully comprehend the causes and clinical implications of imported eosinophilia.

## 5. Conclusions

Eosinophilia is a frequent finding in immigrants from tropical and subtropical regions and represents a useful biomarker of underlying helminth infection. In this cohort, almost half of the patients with eosinophilia remained without an identified etiology despite a structured diagnostic protocol, underscoring the limitations of current approaches. Filariasis, strongyloidiasis and schistosomiasis were the main parasitic causes, and a specific diagnosis was more often achieved in patients with higher eosinophil counts, particularly among those from sub-Saharan Africa. These results highlight the need to reinforce microbiological and parasitological diagnostic capacity, incorporating assays for less common parasites and improving access to advanced tests, in order to reduce diagnostic uncertainty and optimize the care of immigrant populations with eosinophilia.

## Figures and Tables

**Table 1:** Demographic data of patients with eosinophilia stratified by area of origin at the Tropical Medicine Unit, Complejo Asistencial Universitario de Salamanca, Salamanca, Spain, 2008–2023.

	Sub-Saharan Africa, n %		North Africa, n %		Latin America, n %		Asia, n %		All Subjects, n %	
	n = 179		n = 17		n = 123		n = 4		N = 323	
Demographic data										
Mean age ± SD	30.6	±9.6	21.1	±2.6	45.0	±15.2	36.2	±6.5	35.0	±13.6
<18 years	2	1.1	1	5.9	8	6.5	0	0	11	3.4
18–44 years	160	89.4	16	94.1	59	48.0	3	75	232	71.8
>45 years	17	9.5	0	0	56	45.5	1	25	80	24.8
Reason for evaluation										
Screening	148	82.7	16	94.1	52	42.3	1	25	217	67.2
Clinical suspicion	26	14.5	1	5.9	71	57.7	3	75	101	31.3
Other	5	2.8							5	1.5
Laboratory data										
Relative eosinophilia	82	45.8	7	41.2	80	65	2	50	171	52.9
Absolute eosinophilia	97	54.2	10	58.8	43	35	2	50	152	47.1
Syndrome										
Asymptomatic	80	44.7	11	64.7	68	55.3	2	50	161	49.8
Cutaneous	34	19	1	5.9	9	7.3	1	25	45	13.9
Gastrointestinal	42	23.5	4	23.5	15	12.2	0		61	18.9
Respiratory	3	1.7	0		1	0.8	0		4	1.2
Hematologic	4	2.2	0		23	18.7	0		27	8.4
Fever	6	3.4	0		5	4.1	1	50	12	3.7
Ocular	5	2.8	0		1	0.8	0		6	1.9
Genitourinary	0		0		1	0.8	0		1	0.3
Unspecific	5	2.8	1	5.9			0		6	1.9

## Data Availability

The dataset used for this study is available from the corresponding author.

## Supplementary Materials

The following supporting information can be downloaded at https://www.mdpi.com/article/10.3390/tropicalmed11010020/s1, Figure S1: Flowchart of the diagnostic protocol for a patient attending a consultation at the Tropical Medicine Unit, Complejo Asistencial Universitario de Salamanca, Salamanca, Spain, 2008–2023. Figure S2: Geographic distribution of patients included in the study. Tropical Medicine Unit, Complejo Asistencial Universitario de Salamanca, Salamanca, Spain, 2008–2023. Table S1: Clinical manifestations according to the type of confirmed or possible parasitic infection. Tropical Medicine Unit, Complejo Asistencial Universitario de Salamanca, Salamanca, Spain, 2008–2023. Table S2: Symptom frequency according to confirmed or possible parasitic diagnosis. Tropical Medicine Unit, Complejo Asistencial Universitario de Salamanca, Salamanca, Spain, 2008–2023. Table S3: Distribution of confirmed or possible parasitic infections according to degree of eosinophilia. Tropical Medicine Unit, Complejo Asistencial Universitario de Salamanca, Salamanca, Spain, 2008–2023.

## Abbreviations

The following abbreviations are used in this manuscript:

TMU	Tropical Medicine Unit
HIV	Human Immunodeficiency Virus
HBV	Hepatitis B Virus
HCV	Hepatitis C Virus
HTLV	Human T-lymphotropic Virus
