# Supplementary material for: Imported Eosinophilia in Migrants from Endemic Areas in Spain
Source: Trop Med Infect Dis. 2026 Jan 11;11(1):20. doi: 10.3390/tropicalmed11010020 (PMC12846568; doi:10.3390/tropicalmed11010020)
Supplement: Supplementary file 1 [file tropicalmed-11-00020-s001.zip › tropicalmed-3899127-supplementary.pdf]

Supplementary material

Figure S1: Flowchart of the diagnostic protocol for a patient attending a consultation at the Tropical Medicine Unit, Complejo Asistencial Universitario de Salamanca, Salamanca, Spain, 2008—2023.

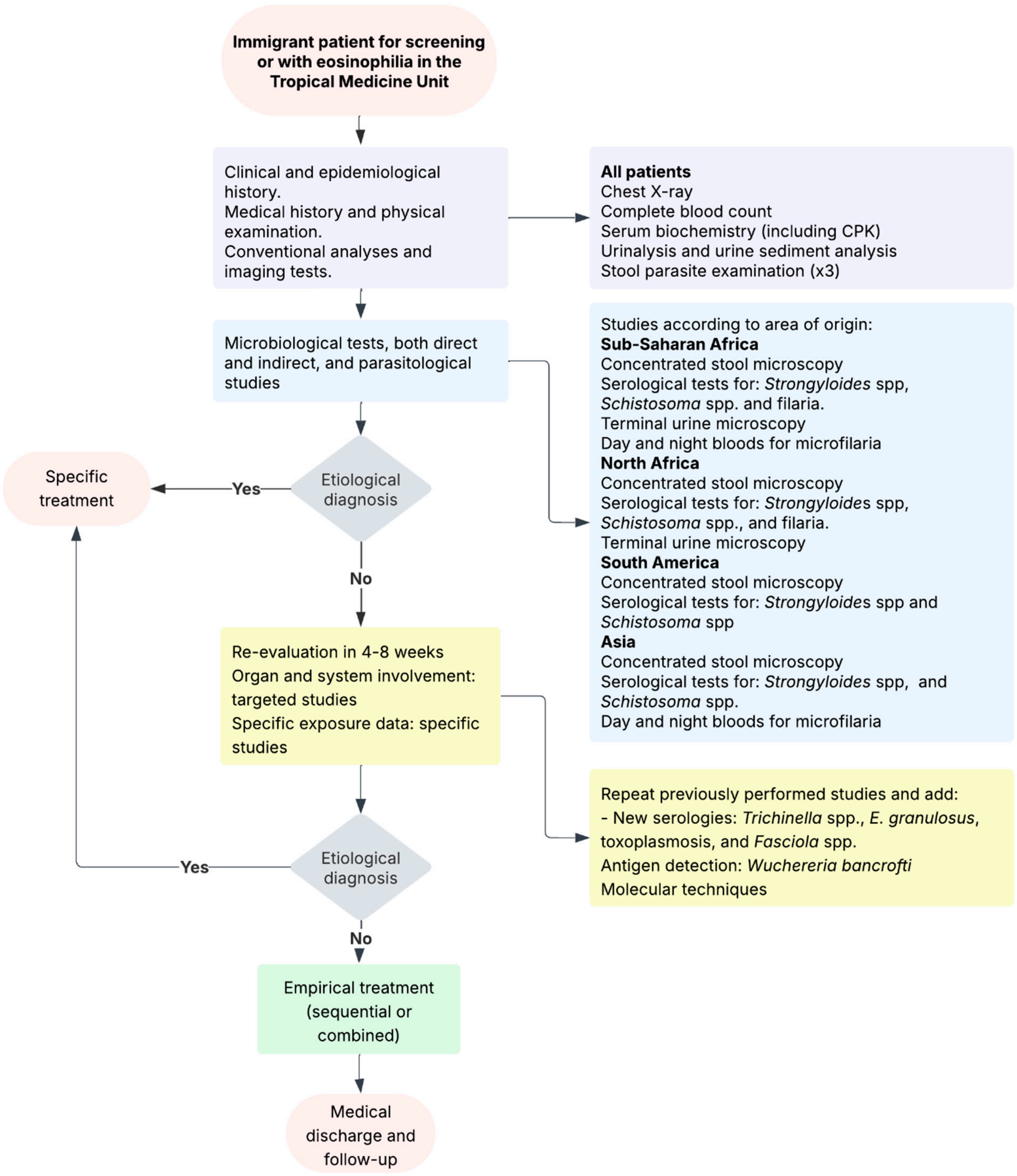

Figure S2: Geographic distribution of patients included in the study. Tropical Medicine Unit, Complejo Asistencial Universitario de Salamanca, Salamanca, Spain, 2008—2023.

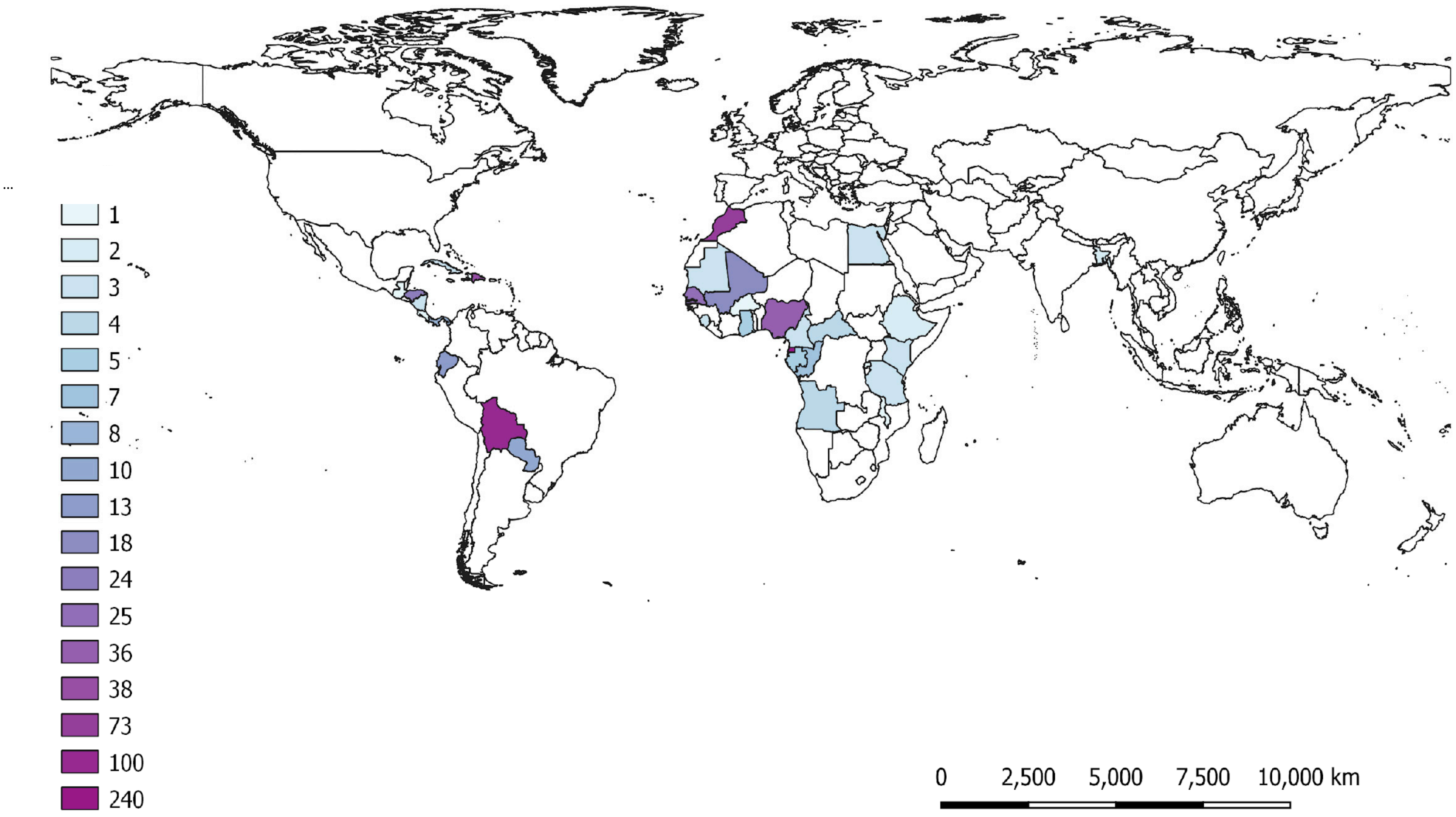

Table S1: Clinical manifestations according to the type of confirmed or possible parasitic infection. Tropical Medicine Unit, Complejo Asistencial Universitario de Salamanca, Salamanca, Spain, 2008—2023.

**Symptoms**	**Schistosomiasis**		**Fasciolosis**		**Strongyloidiasis**		**Filariasis**		**Intestinal parasite**	
	**n**	**%**	**n**	**%**	**n**	**%**	**n**	**%**	**n**	**%**
Asymptomatic	10	45.5	3	37.5	26	41.9	33	55.0	1	14.3
Pruritus	3	13.6	0	0	10	16.1	14	23.3	0	0
Eosinophilia	2	9.1	1	12.5	5	8.1	3	5.0	0	0
Unspecific	2	9.1	0	0	2	3.2	2	3.3	0	0
Abdominal pain	1	4.5	2	25	3	4.8	1	1.7	3	42.9
Abdominal pain and pruritus*	1	4.5	1	12.5	1	1.6	2	3.3	0	0
Cough	1	4.5	0	0	0	0.0	0	0	0	0
Diarrhea	1	4.5	1	12.5	5	8.1	1	1.7	2	28.6
Fever	1	4.5	0	0	4	6.5	1	1.7	0	0
Abdominal pain and diarrhea*	0	0	0	0	2	3.2	0	0	1	14.3
Eye discomfort	0	0	0	0	0	0	3	5.0	0	0
Gastrointestinal bleeding	0	0	0	0	1	1.6	0	0	0	0
Nausea, vomit	0	0	0	0	3	4.8	0	0	0	0

**Each patient appears in a single row. Combined categories (e.g. abdominal pain and diarrhea) indicate both symptoms in the same patient, without double-counting.*

Table S2: Symptom frequency according to confirmed or possible parasitic diagnosis. Tropical Medicine Unit, Complejo Asistencial Universitario de Salamanca, Salamanca, Spain, 2008—2023.

**Symptoms**	**With diagnosis**		**Without diagnosis**	
	**n**	**%**	**n**	**%**
Asymptomatic	73	45.9	84	51.2
Eosinophilia	11	*6.9*	16	9.8
Pruritus	27	17	13	7.9
Abdominal pain	10	6.3	7	4.3
Abdominal pain, pruritus	5	*3.1*	7	*4.3*
Fever	6	3.8	6	3.7
Abdominal pain, Diar	3	*1.9*	5	*3*
Unspecific	6	3.8	4	2.4
Diarrhea	10	*6.3*	4	*2.4*
Nausea, vomit	3	*1.9*	4	2.4
Cough	1	0.6	3	*1.8*
Exanthema	0		3	1.8
Eye discomfort	3	1.9	2	*1.2*
Meteorism	0		2	1.2
Adenitis	0		1	0.6
Eye discomfort, pruritus	0		1	*0.6*
skin lesions	0		1	0.6
Urinary Tract Infect	0		1	*0.6*
Upper Gastrointestinal Bleeding	1	*0.6*	0	

Table S3: Distribution of confirmed or possible parasitic infections according to degree of eosinophilia. Tropical Medicine Unit, Complejo Asistencial Universitario de Salamanca, Salamanca, Spain, 2008—2023.

**Diagnosis**	**Relative eosinophilia** n. % n= 58		**Mild absolute eosinophilia** n. %. n=53		**Moderate absolute eosinophilia** n. % n= 40		**Severe absolute eosinophilia** n. %. n= 8		**All subjects** n. % n =159	
Strongyloides spp + Filarias*	7	12.1	10	18.9	10	25.0	0	0.0	27	17.0
Other coinfections	10	17.2	7	13.2	8	20.0	6	75.0	31	19.5
Positive serological test for schistosomiasis	7	12.1	3	5.7	4	10.0	1	12.5	15	9.4
Positive serological test for strongyloidiasis	13	22.4	14	26.4	5	12.5	0	0.0	32	20.1
Positive serological test for Fasciolosis	6	10.3	2	3.8	1	2.5	0	0.0	9	5.7
Positive serological test for Filariasis*	11	19.0	16	30.2	11	27.5	1	12.5	39	24.5
Intestinal parasite	4	6.9	1	1.9	1	2.5	0	0.0	6	3.8

*Infections classified as 'filariasis' and most coinfections were diagnosed solely by positive serology and should be interpreted as possible infections, which may include some cross-reactive results.
